# Earnings losses in young‐onset dementia: Population‐based study with admin data

**DOI:** 10.1002/alz.14588

**Published:** 2025-02-23

**Authors:** Gaia Bagnasco, Pieter Bakx, Silvan Licher, Job van Exel, Bram Wouterse

**Affiliations:** ^1^ Department of Health Economics, Erasmus School of Health Policy & Management Erasmus University Rotterdam Rotterdam the Netherlands; ^2^ Department of Epidemiology Erasmus Medical Center Rotterdam the Netherlands

**Keywords:** administrative data, data linkage, diagnosis, earnings, employment, event study, social security, young‐onset dementia

## Abstract

**INTRODUCTION:**

Young‐onset dementia is often diagnosed late, leaving gaps in understanding its impact on employment, income, and social security.

**METHODS:**

Analyzing health insurance claims and medical records, we studied 16,010 young‐onset dementia cases and 129,616 matched controls. Using a non‐parametric event study, we assessed earnings, earnings plus benefits, employment losses, and benefit use, considering demographic and socio‐economic factors.

**RESULTS:**

Earnings fell by 58.7% in the years prior to dementia identification, totaling €144,013 in losses, and earnings plus benefits decreased by 20.7% (€68,533). We observed a 35.5 percentage point decrease in employment, a 23.9 percentage point increase in disability insurance, and a 2.7 percentage point rise in welfare benefit use. Primary education and lack of a partner correlated with higher earning losses and lower disability insurance uptake.

**DISCUSSION:**

Early diagnosis and robust social support systems are vital to alleviate the financial and professional challenges faced by individuals with dementia under age 65.

**Highlights:**

- Working‐age persons experience job loss at least 21 years before dementia identification.
- Job loss is linked to 59% (€16,643) earnings drop 16 years before dementia identification.
- Losses in earning were not fully compensated by social insurance.
- A generous social insurance system eases the financial impact of young‐onset dementia.
- Attention is needed in the work environment and on disability benefit decisions.

## 1 BACKGROUND

Young‐onset dementia (YOD), characterized by dementia in individuals under 65 years old, often goes unrecognized initially, with the diagnosis on average being made multiple years after the first onset of symptoms.^1^ Dementia causes diverse neuropsychiatric symptoms that significantly impact work life, resulting in diminished work hours, (voluntary) resignation, and eventual job loss.^2^, ^3^, ^4^, ^5^, ^6^, ^7^, ^8^, ^9^, ^10^ Loss of income for young adults with dementia is a concern as they often face significant financial responsibilities.^7^, ^11^, ^12^ The difficulties in diagnosing dementia at young ages suggest that income and employment effects might start many years before diagnosis. A lack of timely diagnosis might also influence the type and amount of income support individuals receive when unable to continue working. In many countries, different types of social benefits exist for individuals who lose their job due to economic reasons or due to health problems, with the latter generally being more generous but requiring an appropriate medical diagnosis.

Current studies on employment and income effects in individuals with YOD are predominantly qualitative, featuring small samples and descriptions of individuals’ experiences upon leaving employment.^2^, ^3^, ^4^, ^5^, ^6^, ^7^, ^8^, ^9^, ^10^ For instance, interviews with employed individuals experiencing dementia without a formal diagnosis in the Netherlands revealed that insurance physicians struggled to assess work eligibility, resulting in limited or denied disability insurance coverage. When unable to work, the absence of a diagnosis often left only lower welfare benefits as options after unemployment benefits ran out.^13^ Large‐scale quantitative studies on older individuals with dementia have focused on declines in financial capabilities associated with memory impairments among mostly older age groups,^14^ such as irregular bill payments, risky financial decisions, and susceptibility to fraud.^15^, ^16^, ^17^, ^18^, ^19^ Also, trajectories in household wealth for older individuals (aged ≥ 65 years) relative to dementia onset have been examined. The findings indicated that household wealth declined more rapidly among older individuals with dementia compared to controls in the period preceding the onset of dementia.^20^ As most older individuals are above the retirement age and receive fixed pension incomes, these studies have not considered effects on employment and the resulting loss in earnings. While the loss of employment among younger people with dementia might thus be an important issue, quantitative insights into the number of individuals affected and the size of the resulting earning losses are lacking.

In this study, we investigate the association between the onset of dementia before age 65 and employment, earnings, and use of social benefits. To address these questions, we use rich Dutch administrative data for the entire population. We identify individuals with dementia based on health‐care use and analyze their earnings, employment status, and use of benefits up to 21 years prior to the first identification of dementia. We perform a non‐parametric event study comparing persons with dementia to a matched control group from the general population. Subsequently, we assess heterogeneity in the effects across individuals with dementia from different education levels and with and without a partner.

## 2 METHODS

### 2.1 Data sources

For the identification of dementia among adults under the age of 65, we built upon Klijs et al.^21^ and used administrative records on the cause of death and health insurance claims for dementia‐related institutional care eligibility, outpatient medication dispensation, hospital care, mental health care, and home care for the entire Dutch population aged ≥40 and general practitioner care from 10% of the general practitioners.^22^, ^23^, ^24^ Similar methods for identifying dementia have been used in other countries, including the United States, where researchers identified individuals with Alzheimer's disease (AD) by analyzing three types of Medicare claims data: inpatient hospital, institutional outpatient, and physician supplier files.^25^ The data sources, observed between 2016 and 2020, were made available for research by Statistics Netherlands (CBS; see Table S3 in supporting information). This study has been approved according to the governance code of Nivel Primary Care Database, under number NZR‐00321.048. The use of electronic health records for research purposes is allowed under certain conditions. When these conditions are fulfilled, neither obtaining informed consent from patients nor approval by a medical ethics committee is obligatory for this type of observational studies containing no directly identifiable data (arr. 24 GDPR implementation Act ja arr. 9.2 sub j GDPR).

The information on dementia‐related care use was linked to individual‐level data on earnings and earnings plus benefits (covering 2003–2021) and on income sources (1999–2021) through a pseudonymized version of the national identification number. Additionally, demographic details from the municipal register and educational data from educational registries (available for ≈ 50% of the sampled population) were linked. The resulting dataset comprises a single observation per individual for each calendar year.

**RESEARCH IN CONTEXT**

1. **Systematic review**: We reviewed the literature using traditional resources (e.g., PubMed). While many publications have examined the decline in financial capabilities and income among older individuals with dementia, there still exists a notable gap in understanding the financial implications prior to dementia identification, especially concerning earnings and income losses, employment trends, and benefit receipt among individuals under age 65.
2. **Interpretation**: Our findings represent the first population‐level longitudinal evidence showing that declines in cognitive impairment affect professional functioning and are associated with financial hardships for individuals under age 65 with dementia in the Netherlands, particularly in the years before dementia is identified and dementia care is received. This underscores the critical role of a strong safety net in alleviating the financial burdens faced by these individuals.
3. **Future directions**: Future research on potential disparities in disability insurance provision preceding a dementia diagnosis would be a valuable addition to this field of study.

### 2.2 Study population

#### 2.2.1 Dementia cases

**FIGURE 1:**
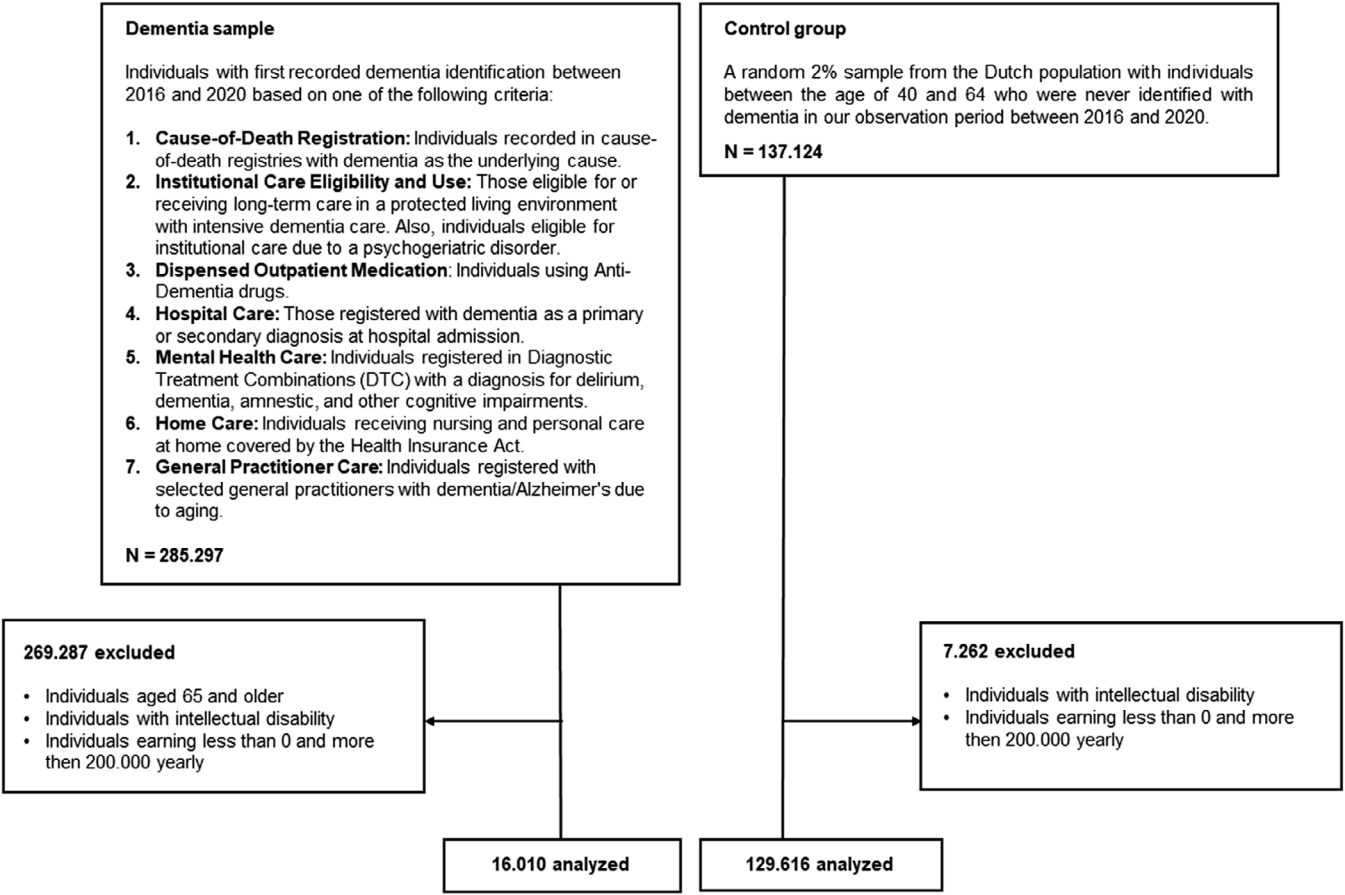
Flowchart sample selection (dementia sample and control group).

We included individuals aged 40 to 64 with their first recorded dementia‐related care use between 2016 and 2020 (Figure 1). The coverage in our study extended to the entire Dutch population in this age group, with the notable exception of general practitioner health records, which were available for 10% of the non‐institutionalized population. Table S3 displays a detailed overview of the sources, definitions, and classifications related to dementia identification. We excluded individuals eligible for institutional care due to an intellectual disability, as they often follow distinct labor market trajectories, and individuals reporting negative earnings or earnings > €200,000 in at least one calendar year to reduce noise caused by significant outliers.

#### 2.2.2 Control group

We drew a 2% random sample from the Dutch population, comprising individuals aged 40 to 64, who did not receive dementia care between 2016 and 2020. To ensure comparability to the YOD sample, the control group was adjusted using sampling weights. For each outcome, weights were defined such that the sex, age, and outcome distribution in the base year (the first available observation year) was equal in the control and the dementia group. Notably, one set of weights was computed using earnings in 2003, another relied on earnings plus benefits in 2003, and the final set was derived from employment status in 1999 (see Table S4 in supporting information). Individuals with an intellectual disability and individuals reporting negative earnings or earnings > €200,000 in at least one calendar year were also excluded from the control group.

### 2.3 Outcomes

Outcome measures encompassed (1) earnings, (2) earnings plus benefits, and (3) income source, categorized as employment, disability insurance benefits, welfare benefits, unemployment benefits, retirement benefits, or other social security benefits. Earnings are equal to an individual's gross income from employment and self‐employment (euros [€], 2021 price level). Earnings plus benefits refer to an individual's personal income, which includes earnings along with additional income from social insurance benefits. Employment encompasses both employees and self‐employed individuals.

Disability insurance benefits refer to those receiving sickness or disability benefits in the reporting year. Sickness benefits are intended to cover short‐term illnesses or injuries. In the Netherlands, they amount to usually 70% of the daily wage and are provided for a period of a maximum of 2 years before disability benefits can be applied for.^26^ Throughout this designated “waiting period,” employers are tasked with facilitating the worker's return to work by providing reintegration services or accommodations, alongside maintaining wage payments.^27^

After sickness benefits, individuals can apply for disability benefits, which are intended to provide ongoing support for individuals with long‐term or permanent disabilities. In the Netherlands, eligibility for disability insurance is determined through a medical assessment and an evaluation of remaining job opportunities by a physician. A documented diagnosis is required to receive benefits. Depending on the assessed “remaining work capacity,” benefits can range up to 75% of the last earned gross income, and last until reaching retirement age.^26^

In cases of involuntary dismissal, individuals in the Netherlands can access unemployment benefits for a period ranging from 3 to 24 months depending on their work history. Initially, these benefits amount to 75% of the last salary for the first 2 months, and 70% thereafter, contingent on actively seeking new employment.^28^ If reemployment proves elusive after exhausting unemployment benefits, individuals may apply for welfare benefits.

Welfare benefits refer to social assistance and are a financial benefit provided to individuals who lack sufficient income or assets to meet their basic living expenses, with the amount determined by the established social minimum, which is ≈ €1050 per month in the Netherlands.^29^

All income sources were treated as separate dichotomous variables. Further details on the definitions of outcome measures can be found in Tables S5‐S6 in supporting information.

### 2.4 Sociodemographic characteristics

Sociodemographic characteristics comprised age, sex, migrant status, partner status, and education. Age is measured at a yearly level. Migrant status is determined by the individual's and their parent's birthplace, categorized into Western regions (Europe, North America, Oceania, Indonesia/Japan) and non‐Western regions (Africa, Latin America, Asia, including Turkey). Partner status concerns marital status as derived from commitment data recorded in the Personal Records Database (BRP), indicating the formal position of an individual with reference to marriage and registered partnership. Observations were further stratified by four educational levels: (1) missing values, (2) primary education, (3) secondary education, and (4) higher education, with definitions available in Table S7 in supporting information.

### 2.5 Statistical analysis

We estimated changes in earnings, earnings plus benefits, and income source in the years before and after the identification of dementia using a non‐parametric event study. We regress outcome measures on time‐to‐event dummies for a sample of individuals who experience an event within the observation period, albeit at different points in time.^30^ Based on the available data, we included observations for earnings and earnings plus benefits over the years 2003 to 2021, and for income sources over 1999 to 2021, for individuals identified with dementia in the years 2016 to 2020. This resulted in an unbalanced panel, where earnings and earnings plus benefits were tracked from a maximum of 17 years before to 1 year after the year dementia was first identified, and income sources were followed from a maximum of 21 years prior to and 1 year after dementia identification. To separate the effects of calendar year and age from the effect of the time to the identification of dementia, we included the control group of individuals not receiving dementia care, adjusted with sampling weights to ensure comparability.

We estimated separate models for each outcome. Each model was a linear regression with time‐to‐event dummies, where time‐to‐event was defined as the number of years prior to (or after) the identification of dementia. For the control group, all time‐to‐event dummies were zero by construction. We further included calendar year dummies, age, sex, migrant categories, partner status, and education categories. The main results of interest were the coefficients of the time‐to‐event dummies, which show the development of the outcomes in the 17 or 21 years prior to and 1 year after the identification of dementia compared to a control group with similar characteristics who did not use any dementia‐related care during the observation period.

For our heterogeneity analyses, we explored whether associations differed among persons with YOD based on education, partner status, age, and nursing home eligibility. Education has been suggested to be a protective factor against dementia^31^, ^32^, ^33^, ^34^ and presents a strong positive relationship with earnings,^35^ while a partner may be a source of both social and financial support, including assistance in managing finances and ensuring financial stability^36^ and a partner's income has consequences for welfare benefit eligibility. For the sensitivity analysis, individuals with and without nursing home eligibility were compared as a proxy for dementia severity to explore variations in the disease's financial impact. All analyses were performed with STATA (version 16).

## 3 RESULTS

### 3.1 Study sample

We identified 16,010 individuals with dementia under the age of 65 in medical records and health insurance claims, closely aligning with the estimated 14,000 to 17,000 YOD persons in the Netherlands from prior research.^37^ Moreover, we selected 129,616 matched controls. Table 1 compares the YOD sample to the control sample on the demographic characteristics and outcome measures in the first year in which the relevant outcome measure was observed (1999 for income sources and 2003 for earnings and earnings plus benefits), and the first year dementia care was observed (2016).

**TABLE 1:** Average sample characteristics of identified dementia cases and matched control group.

	Dementia cases (*n* = 16,010)		Control group (*n* = 129,616)	
Characteristics	2003	2016	2003	2016
Age, mean [standard deviation]	42.5 [5.9]	55.4 [6.0]	42.0 [7.0]	54.6 [7.3]
Female, %	45.4	45.3	45.4	45.3
Western region %	87.7	86.7	88.5	87.3
w/ partner, %	54.9	46.7	66.3	64.2
Education level, %				
* Primary education*	24.1	25.0	16.8	17.2
* Secondary education*	19.1	19.0	18.4	18.4
* Higher education*	10.2	10.0	13.3	13.1
* Missing value*	46.5	46.0	51.5	51.3
Annual earnings in EUR, mean [standard deviation]	28,906 [32,402]	15,717 [28,101]	30,491 [34,744]	30,694 [36,148]
Annual earnings plus benefits in EUR, mean [standard deviation]	30,190 [22.674]	28.009 [22.383]	30,817 [24,405]	34,384 [27,718]

	1999	2016	1999	2016
Employment, %	67.1	37.5	67.1	68.7
Disability insurance benefits, %	17.3	37.4	8.4	12.5
Welfare benefits, %	13.8	17.0	6.7	7.1
Unemployment benefits, %	4.9	6.7	3.4	7.4
Retirement benefits, %	0.7	21.4	0.6	16.9
Other social security benefits, %	5.1	10.9	2.1	4.2

*Note*: Average weighted sample characteristics for individuals with dementia under age 65 and matched control group compared for the first outcome observation year (1999 for income sources or 2003 for earnings and earnings plus benefits) and the first dementia observation year (2016). For the demographic characteristics and earnings, sampling weights were applied based on sex, age, calendar years, and earnings in 2003. For earnings plus benefits, sampling weights based on sex, age, calendar years, and earnings plus benefits in 2003 were applied. For income sources, sampling weights based on sex, age, calendar years, and employment status in 1999 were applied. The differences between the dementia sample and the control group were statistically significant for all characteristics in both the initial observation year and 2016, as determined by *t* tests, except for sex in 2003 and 2016 and employment in 1999.

After applying sampling weights, the demographic characteristics of both samples were found to be comparable, with three notable exceptions. First, individuals in the dementia sample less frequently reported having a partner, both in 2003 and 2016. Also, young individuals with dementia were more likely to receive disability insurance benefits and welfare benefits compared to the control group, both in 1999 and 2016. These differences were statistically significant, as confirmed by *t* test analyses.

### 3.2 Earnings and earnings plus benefits

**FIGURE 2:**
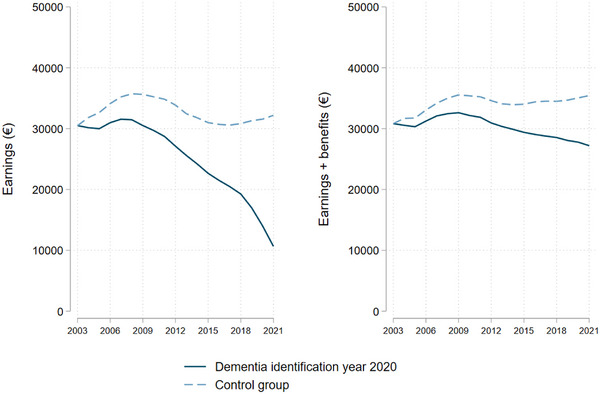
Earnings and earnings plus benefits between 2003 and 2021 for individuals with dementia (identified in 2020) and the control group. Note: Earnings and earnings plus benefits over all observation years (2003 and 2021) in which a distinction is made between individuals identified as a person with dementia in the year 2020 and the control group. For the control group sampling weights have been applied to make this group more comparable to our dementia group. The weights were established based on sex, age groups, and calendar years. Also, one set of weights is computed using earnings in 2003 (figure on the left) and another relies on earnings plus benefits in 2003 (figure on the right).

For individuals identified with dementia, earnings and earnings plus benefits declined over the study period. In 2007, the highest annual earnings were recorded at €31,549, but by 2021, this amount had dropped to €10,651. Similarly, the highest annual level of earnings plus benefits for this group was €32,619 in 2009, which decreased to €22,365 by 2021 (see Figure 2). The control group also experienced a reduction in both earnings and earnings plus benefits, particularly noticeable during the period surrounding the financial crisis (2008–2011). However, the magnitude of this decline was less pronounced. Only between 2008 and 2017 earnings dipped, while earnings plus benefits experienced a decline between 2010 and 2014.

**FIGURE 3:**
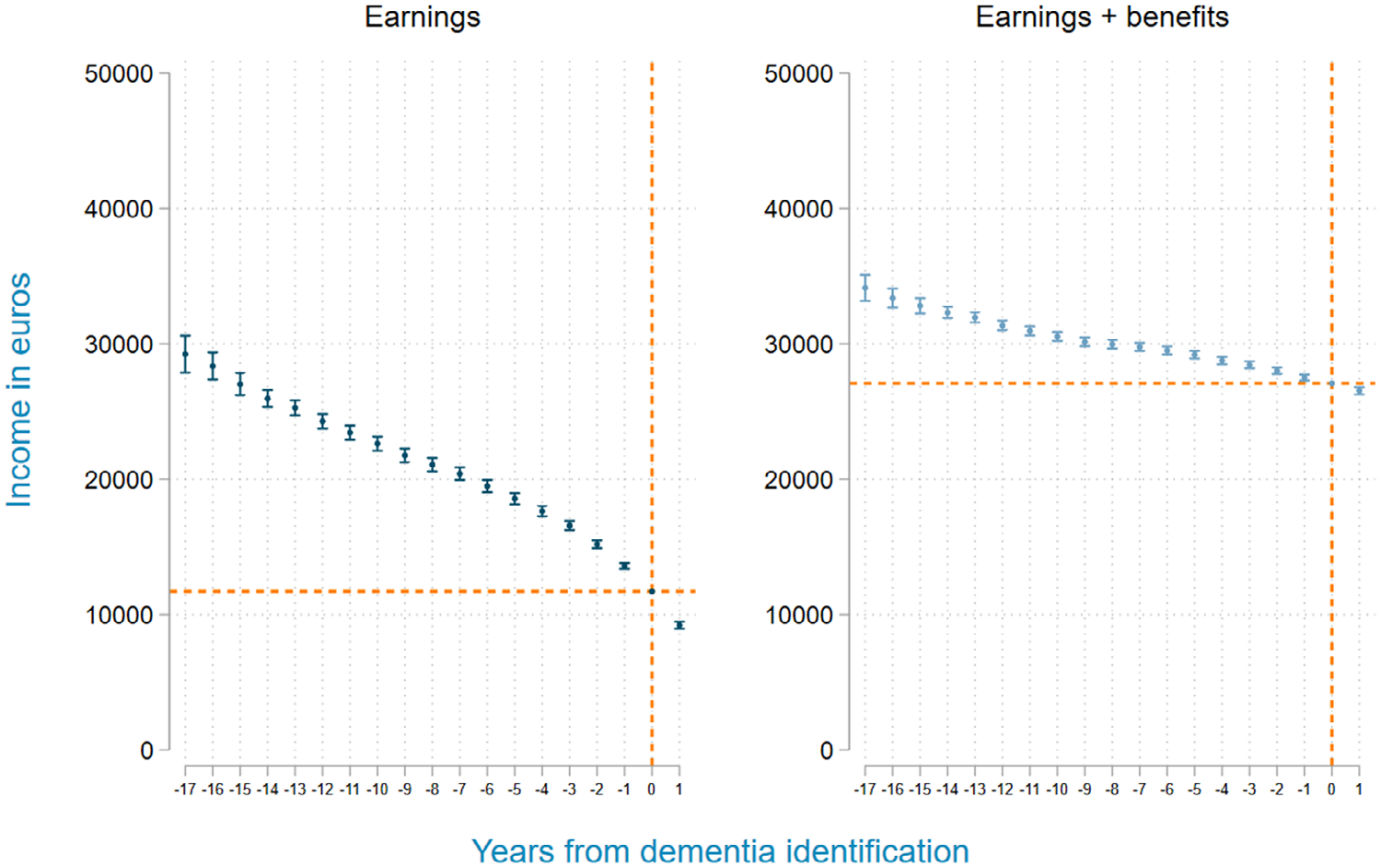
Earnings and earnings plus benefits in the years prior to the identification of dementia. Note: Regression results of linear regressions for earnings and earnings plus benefits with time‐to‐event dummies. Time to event was defined as the number of years prior to and after the identification of dementia. For the control group, all time‐to‐event dummies were zero by construction. We further included calendar year dummies, age, sex, migrant categories, partner status, and education categories. The results presented in this figure are the sum of the coefficients of the time‐to‐event dummies and the mean in earnings or earnings plus benefits at identification. This shows the development of the outcomes in the 17 years prior to 1 year after the identification of dementia compared to a control group with similar characteristics who did not use any dementia‐related care during the observation period. Sampling weights were applied based on sex, age groups, and calendar years to make the control group more comparable to the dementia group. One set of weights is computed using earnings in 2003 (figure on the left) and another relies on earnings plus benefits in 2003 (figure on the right).

The results of the regression analyses confirm this trend (Figure 3): the earnings and earnings plus benefits of individuals with dementia decline, relative to the control group, in the years before the first dementia identification. Sixteen years before the identification of dementia, these individuals earned €16,643 more annually (58.7% of the mean income in the year of dementia identification), while earnings plus benefits were €7052 (20.7%) higher 17 years before identification. Cumulatively, over all the observation years leading up to identification, individuals with dementia experience a loss of earnings of €144,013 compared to the control group, and a loss of earnings plus benefits of €68,533.

### 3.3 Income sources

**FIGURE 4:**
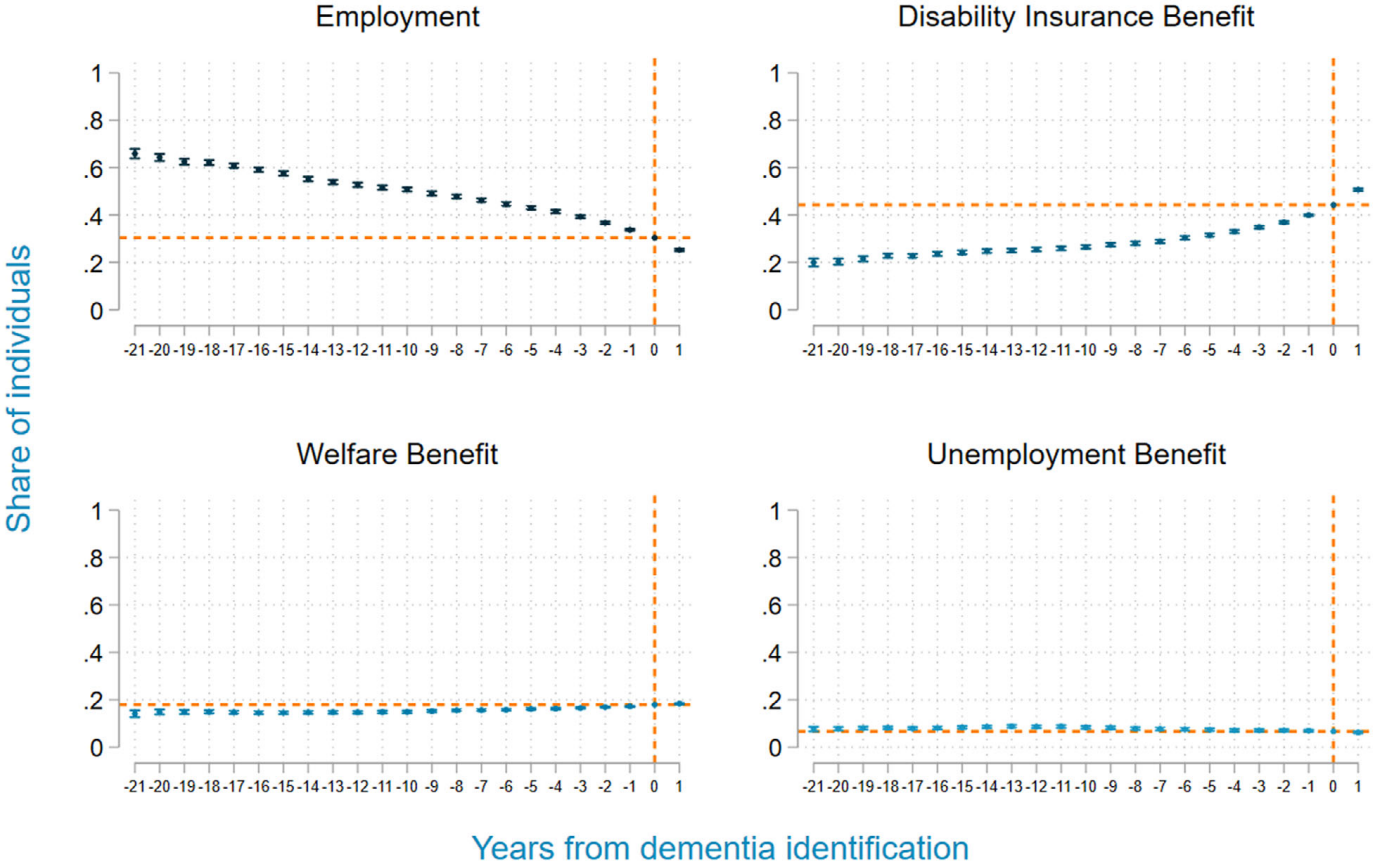
Share of individuals with dementia using different income sources in the years prior to the identification of dementia. Note: Regression results of linear regressions for employment, disability insurance benefits, welfare benefits, and unemployment benefits with time‐to‐event dummies. Time to event was defined as the number of years prior to and after the identification of dementia. For the control group, all time‐to‐event dummies were zero by construction. We further included calendar year dummies, age, sex, migrant categories, partner status, and education categories. The results presented in this figure are the sum of the coefficients of the time‐to‐event dummies and the mean in earnings or earnings plus benefits at identification. This shows the development of the outcomes in the 21 years prior to 1 year after the identification of dementia compared to a control group with similar characteristics who did not use any dementia‐related care during the observation period. Sampling weights were applied based on sex, age groups, and employment status in 1999 to make the control group more comparable to the dementia group.

Figure 4 illustrates the share of individuals with dementia using different income sources in the years prior to the identification of dementia. As indicated, employment levels dropped by 35.5 percentage points (54.2% of baseline level). Wald tests comparing coefficients to the coefficient for the earliest period reveal that the decline begins at least as early as our initial observation year, which is 21 years prior to the year of dementia identification. This implies that employment losses might potentially commence even earlier than this timeframe. Additionally, there was a 23.9 percentage point increase (or 118.9% of baseline level) in the share of individuals using disability insurance benefits starting 20 years before identification and a 2.7 percentage point increase of welfare benefit users (17.6% of baseline level) starting 9 years before identification. Finally, a 1.7 percentage point (19.5% of baseline level) decrease in unemployment benefits was observed. This decrease started 15 years prior to the year of the first dementia identification.

### 3.4 Heterogeneity

Observable declines in earnings and earnings plus benefits relative to the respective means at identification were evident across all education levels (Table 2). Individuals with primary and secondary education both experienced significant earning losses in relative terms. Primary‐educated individuals saw a 61.8% relative decline (€7427) starting 13 years before identification, while secondary‐educated adults experienced a 62.4% relative decline (€15,745) beginning 15 years before identification. In contrast, higher educated individuals faced a 54.6% relative decrease (€24,912) commencing 14 years before identification. In earnings plus benefits, higher educated individuals experienced the most substantial relative decline, at 18.5% (€9011) starting 13 years before identification. This exceeded the losses for primary‐educated individuals, with an 8.2% relative decrease (€1704) starting 12 years before identification.

**TABLE 2:** Relative difference in earnings, earnings plus benefits, and income sources for different education levels and individuals with and without a partner.

	Relative difference Earnings (%)	Absolute difference Earnings (€)	Relative difference Earnings plus benefits (%)	Absolute difference Earnings plus benefits	Relative difference Employment (%)	Absolute difference Employment (pp.)	Relative difference Disability insurance benefit (%)	Absolute difference Disability insurance benefit (pp)	Relative difference Welfare benefit (%)	Absolute difference Welfare benefit (pp)
**Primary education** **(*N* = 3954)**	61.8	€7427 [6625–8229]	8.2	€1704 [1215–2193]	57.9	24.8 [22.7–26.8]	−49.8	−12.3 [–13.9–10.7]	−1.5	−0.6 [–1.1–0.1]
**Secondary education** **(*N* = 3014)**	62.4	€15,745 [14,123–17,367]	16.3	€4912 [4176–5649]	53.4	35.5 [33.3–37.7]	−121.2	−26.3 [–28.4–24.3]	−34.8	−4.9 [–6.4–3.5]
**Higher education** **(*N* = 1610)**	54.6	€24,912 [22,156–27,669]	18.5	€9011 [7370–10,652]	43.6	32.5 [29.7–35.3]	−196.8	−30.5 [–33.2–27.8]	−15.8	−1.5 [–2.4–0.6]
**Individuals with partner** **(*N* = 6750)**	53.3	€16,976 [15,559–18,392]	17.2	€6100 [5087–7113]	41.2	28.0 [25.1–31.0]	−109.0	−21.9 [–24.5–19.2]	−30.0	−3.0 [–4.4–1.6]
**Individuals without partner** **(*N* = 3719)**	68.2	€17,070 [15,238–18,902]	20.7	€6366 [5504–7228]	54.9	35.4 [32.2–38.7]	−46.6	−12.4 [–14.9–10.0]	−8.8	−2.1 [–3.2–1.0]

*Note*: This analysis examines the average relative difference and absolute difference in outcome measures across primary, secondary, and higher education; individuals with a partner; and individuals without a partner. The absolute difference is defined as the difference between the mean of the outcome measure at identification and the corresponding value at the starting point of significant change (K years before identification), expressed in percentage points for each relevant subgroup. The relative difference equals the change between the starting point and the mean of the respective subgroup in the dementia identification year (K = 0) divided by the sum of that mean and coefficient in the starting point. The starting point represents the event year (K) in which the coefficient of the outcome measure (earning, earnings plus benefits, and income source) is significantly different from the first observed event year (K = –17 for earnings and earnings plus benefits; K = –21 for income sources).

Primary‐educated individuals faced the largest relative declines in employment, experiencing a 57.9% decrease (24.8 percentage points) starting 17 years before identification. Higher ‐educated individuals also experienced a relative decline, though less pronounced, at 43.6% (32.5 percentage points) starting 15 years before identification. For disability insurance benefits, the uptake was also the smallest over time for primary‐educated individuals, with only a 49.8% relative increase (12.3 percentage points) commencing 11 years before identification. In welfare benefits, the highest relative increase in use was observed among individuals with secondary education, with a substantial 34.8% rise (4.9 percentage points) starting 13 years before identification. Comparatively, those with higher education saw a 15.8% increase (1.5 percentage points) beginning 3 years prior to identification, while individuals with primary education had a more modest 1.5% rise (0.6 percentage points) starting 1 year before identification.

Individuals without a partner faced a 68.2% loss (€17,070) in earnings starting 17 years prior to identification compared to a 53.3% relative reduction (€16,976) beginning 16 years before identification for those with a partner. Also, larger relative declines in earnings plus benefits were found for individuals without a partner 16 years before identification. These individuals were confronted with a 20.7% relative loss (€6366), while those with a partner experienced a 17.2% relative reduction (€6100) in earnings plus benefits 16 years before identification. Furthermore, individuals without a partner encountered the largest relative employment losses (54.9% vs. 41.2%) and the lowest relative disability insurance benefits receipt (46.6% vs. 109.0%).

## 4 DISCUSSION

### 4.1 Main findings

This nationwide study presents three key findings about changes in income and income sources among individuals with YOD prior to the identification of the disease. First, individuals under the age of 65 with dementia experienced employment losses starting 21 years before the first year of identification of dementia. These job losses were accompanied by declines in annual earnings equal to 58.7% (€16,643) over a 16‐year period. Second, our results reveal that a generous social insurance system indeed protects many individuals against the financial impact of dementia at a younger age. The comparison between the loss in earnings and in earnings plus benefits shows that an important part of the loss in earnings from work is compensated by social insurance. Third, individuals with primary education and individuals without a partner are faced with large relative earning and employment losses and the lowest relative uptake in disability insurance benefits. These findings underscore the complex challenges faced by specific demographic groups in mitigating the financial impacts of dementia.

### 4.2 Comparison to literature

Earlier qualitative studies have highlighted that younger individuals with dementia experienced job losses in the years leading up to their diagnosis,^2^, ^3^, ^4^, ^5^, ^6^, ^7^, ^8^, ^9^, ^10^ resulting in significant financial challenges.^7^, ^11^, ^12^ However, these studies did not provide insight into the number of individuals affected in the population and the size of the employment and income losses. This study is the first to examine and quantify these within an entire population of individuals with dementia under age 65. Prior work using administrative data for the entire population with Medicare beneficiaries who had one or more claims with a diagnostic code indicating AD and related dementias (ADRD) has mainly focused on the older population in the United States, at ages when most individuals have already retired.^14^ Nicholas et al.^14^ found that older individuals with dementia experience problems in financial decision making, such as missing credit account payments, as early as 6 years before the identification of dementia; Li et al.^20^ found that household income declined 8 years before the onset of dementia for older persons. Our study demonstrates the value of extending these approaches beyond the traditional target group of older adults with dementia to younger individuals of whom many are still active in the labor market. Like these studies, we observe an impact many years before the identification of dementia. We demonstrate that the social insurance system protects many individuals against the financial impact of dementia at a younger age, yet the social safety net is not without holes. YOD individuals on average lose 20.7% (€7052) in earnings plus benefits in the 17 years prior to identification of dementia. The fact that we find a 17.6% relative increase in welfare benefit receipts suggests that for some of the younger individuals the dementia‐related health problems, which would potentially qualify them for (higher) disability insurance benefits, are not detected quickly enough.

Furthermore, considering the selection of outcome measures, Li et al.^20^ focused on household income, which might differ from the income declines for individuals with dementia themselves; dementia might also influence the income of other household members as they either start working more to compensate^38^ or less to take care of their family member. We examined the earnings and earnings plus benefits of the person with dementia to focus on the direct income effects. Additionally, we note that individuals without a partner undergo more pronounced declines compared to those with a partner, suggesting that even for the individual income partners may play a crucial role in mitigating the adverse financial consequences for individuals with dementia. As partners often are the first to notice symptoms,^39^, ^40^, ^41^ individuals without a partner potentially experience larger delays in diagnosis, leading to a delayed adjustment in work or financial planning and thereby larger earnings and earnings plus benefits losses. Moreover, earning losses are potentially mitigated, as partners may assist individuals with dementia by helping them adapt to functional losses. Prior research suggests that partners can identify retained abilities and implement strategies that maximize these skills,^42^ which may help employed individuals with dementia stay in the workforce longer and delay earning losses.

### 4.3 Strengths and limitations

This research improves our comprehension of the financial aspects of dementia and the related factors. It accomplishes this by using extensive real‐world data, which addresses significant methodological constraints present in small‐scale qualitative studies. We were able to observe a unique long observation period, spanning 17 years before identification for earnings and even 21 years for income sources. Furthermore, unlike earlier studies, we used a representative population derived from yearly recorded administrative records on the cause of death and health insurance claims. Additionally, we compared the data of people with dementia to that of a matched control group without dementia‐related care usage. Moreover, there were no missing outcomes, as we used annual data from national registries. Finally, the Dutch setting, with universal health insurance, means that dementia diagnoses are not missed or delayed if losing one's job leads to changes in health insurance, as may be the case in the United States. This eliminates the confounding effect of health insurance loss on dementia diagnosis.

Nevertheless, we also highlight six main limitations that should be taken into account when interpreting study findings. First, we observe dementia diagnoses only on the basis of health insurance claims for dementia care and medical records, meaning that we have no information on the exact timing of the diagnosis, the subtype, and the progression of the disease. As we likely identify people in the years after the initial diagnosis, the observation period likely includes some years during which part of the sample was already diagnosed. Despite this limitation, we observe that, depending on the outcome, the decline starts 12 to 18 years before the dementia identification year and given the average life expectancy of 10 years after receiving the diagnosis,^43^ it is unlikely that the diagnosis was set an equally long period before the onset of dementia care and thus the losses in income and employment precede the diagnosis by at least a few years.

Second, confounding is a possible issue. Prior to applying sampling weights to the control group, there were disparities in earnings and earnings plus benefits levels between the YOD sample and the control sample even 17 years before the year of dementia identification. Both groups were possibly different in terms of other, unobserved characteristics and hence on different paths for income and employment. We tried to mitigate these differences by applying sampling weights using information from the earliest data point available.

Third, one of our main findings is that individuals under age 65 with dementia experience employment losses 21 years before identification. The finding that there are differences in the earliest year that we observe suggests that the difference may start even earlier.

Moreover, we found that disability insurance benefit receipt began increasing as early as 20 years before dementia diagnosis. Research indicates dementia‐related changes can appear well before symptoms are recognized, with cognitive decline observed 18 years prior to AD diagnosis among older adults and biological changes up to 20 years earlier in individuals genetically predisposed to young‐onset AD.^44^, ^45^

Diagnosing YOD is challenging due to its diverse causes, including AD, frontotemporal, and vascular dementia; traumatic brain injury; and alcohol‐related dementia.^46^ Moreover, young‐onset AD often presents with atypical symptoms such as seizures and muscle stiffness rather than the memory loss commonly associated with dementia.^46^ Furthermore, initial symptoms are frequently misattributed to psychiatric disorders, depression, or other neurological conditions, resulting in frequent misdiagnoses.^47^ In the Netherlands, disability insurance benefits require a documented diagnosis by an examining physician. However, we do not have data on the specific diagnoses recorded during this period; other conditions may have been documented to justify eligibility.

Furthermore, there are no data on retirement benefits for individuals aged < 55 for the period before 2009, meaning we underestimate retirement rates for this age group in these years. Data from the period after 2009 indicate that this group is very small: 13.1% of those aged < 55 are retired.

Finally, regarding external validity, it is important to acknowledge that the findings may have some restrictions in terms of generalizability due to variations in health care and social support systems across countries. While the Dutch social insurance system may largely protect younger individuals with dementia, its applicability to other nations with different systems and safety nets should be considered when interpreting the results.

## 5 CONCLUSION

The results underscore the substantial negative impact of cognitive impairment on professional functioning and the associated financial hardships for individuals under the age of 65 with dementia in the Netherlands, particularly in the years before dementia is identified and dementia care is received. This shows the critical role of a strong safety net in alleviating the financial burdens faced by these individuals. Also, a prompt diagnosis plays a pivotal role in ensuring the effective functioning of social insurance systems, especially in facilitating suitable compensation for employment setbacks via disability insurance benefits, particularly for the most vulnerable groups.

## CONFLICT OF INTEREST STATEMENT

None declared.

## CONSENT STATEMENT

Informed consent was not necessary for this study.

## Supporting information



Supporting Information

Supporting Information
