# Supplementary material for: Earnings losses in young‐onset dementia: Population‐based study with admin data
Source: Alzheimers Dement. 2025 Feb 23;21(2):e14588. doi: 10.1002/alz.14588 (PMC11847646; doi:10.1002/alz.14588)
Supplement: Supplementary file 1 — Supporting Information [file ALZ-21-e14588-s002.docx]

**Appendix A – Dementia identification factors**

**Supplemental Table 3. Identification of sample of individuals with dementia**

| **Dementia identification item** | **Data source** | **Dementia identification code** |
| --- | --- | --- |
| Cause-of-death registration | Cause-of-death statements of deceased individuals are completed by physicians and registered by Statistics Netherlands using the 10th edition of the International Classification of Diseases (ICD) system. The registration provides information about the underlying causes of death of individuals. Additionally, for those who have died due to non-natural causes, it also includes the main injury and the location of the accident. | References to dementia as the underlying cause of death were selected for this study. These are individuals registered with the following ICD-10 diagnostic codes: F00 (Dementia in Alzheimer’s disease), F01 (Vascular dementia), F02 (Dementia in other diseases classified elsewhere), F03 (Unspecified dementia), or G30 (Alzheimer’s disease). |
| Institutional care eligibility and use | The Care Needs Assessment Center (i.e., CIZ) conducts care needs assessments and grants long-term care eligibility to establish a person's eligibility for long-term care.  Long-term care use is registered by the Dutch Central Administration office (i.e., CAK). They register care packages (ZZP) that describe the type and intensity of institutional care used. | Individuals who are eligible for or receive long-term care in a protected living environment with intensive dementia care (care package “ZZP VV5”) and individuals who are eligible for institutional care because of a psychogeriatric disorder were selected. |
| Dispensed outpatient medication | The Dutch National Health Care Institute (i.e., Zorginstituut Nederland) registers all dispensed outpatient medications that are reimbursed under basic healthcare insurance. The medication is aggregated according to the Anatomical Therapeutic Chemical (ATC) classification drawn up by the World Health Organization (WHO), level 4. | Individuals using N06D (Anti-Dementia drugs) medication were selected. |
| Hospital care | Hospital care with reported diagnoses is tracked in all general and academic Dutch hospitals as well as three classified hospitals (two cancer clinics and one eye hospital). Conditions are diagnosed and documented during daycare admissions, inpatient admissions, and "long-term observations without an overnight stay". Diagnoses are coded with the ICD-10 classification system, where a distinction between the primary and secondary diagnosis is made in the admission recording. | Patients hospitalized in the listed Dutch hospitals with ICD-10 diagnosis codes F00, F01, F02, F03, and G30 were selected. |
| Mental health care | Mental health care is registered with the use of Diagnostic Treatment Combinations (DTCs) in which diagnoses, treatments, and costs are recorded. When categorizing these DTCs according to the mental disorders that are treated, ICD-10 codes are used. | Individuals with a diagnosis of delirium (F05), dementia (F00-F03), amnestic, and other cognitive impairments (F04) based on the ICD-10 codes. The category F04 encompasses cognitive disorders that do not meet the criteria for dementia but still have a significant impact on memory and other cognitive functions. |
| Home care | Information on nursing and personal care provided at home that is covered by the Health Insurance Act is collected by Vektis. | Home care for individuals with dementia is based on four indicators. These indicators include:   - Care for vulnerable elderly and chronically ill individuals lasting longer than 3 months (psychogeriatric/psychiatric) - Dementia care network. This is a form of care in which various healthcare providers and organizations collaborate to enhance the care for people with dementia and their caregivers. - PG care. This refers to the care and treatment of psychogeriatric patients in a nursing home or long-term care facility. It encompasses the specific care and support required for individuals with dementia or other mental health conditions in the elderly. - PG complex care. This refers to a specific type of psychogeriatric care that involves complex care needs.   These diverse indicators are recorded using “care type codes”, which denote a specific category or type of care provided by healthcare providers or caregivers. These codes are utilized in the AGB register (General Data Management for Healthcare Providers) to register and identify healthcare providers and caregivers.  For the four indicators, the following care type codes are used:   - Care for vulnerable elderly and chronically ill individuals, lasting longer than 3 months (psychogeriatric/psychiatric): Care Type 42 - Dementia care network: Care Type 98 - PG (Psychogeriatric) care: Care Type 42 - PG complex care: Care Type 42 |
| General Practitioner care | Information from a sample of Dutch general practitioners is observed. For general practitioners, the International Classification of Primary Care (ICPC) is accepted as the standard for coding and classifying complaints, symptoms, and conditions. | Individuals registered at the selected general practitioners with the code P70 (Dementia/Alzheimer’s due to ageing) were selected. |

**Appendix B - Detailed explanation of constructed sampling weights**

In the empirical analysis, different sampling weights are employed for each outcome: earnings, earnings plus benefits and source of income. The purpose of these weights is to create a control group that is similar in terms of characteristics in the base year to the group of individuals who are identified with dementia. As the base year, we use the first year of observation, which is 2003 for earnings and earnings plus benefits, and 1999 for employment. In each observation year *t*, we stratify the dementia- and the control group based on the values of the relevant outcome and a set of covariates *in the base year t_0_*. Then, we weigh the observations in each stratum within the control group so that the total number of observations is equal to the number of people in the dementia group. Let $D_{t}^{s}$ be the number of observations within stratum *s* in the dementia group in year *t*, with membership of *s* being determined by the characteristics of each individual in *t_0_*. $C_{t}^{s}$ is the number of individuals in the control group in the same stratum and year. Then the weight for all individuals in the control group in stratum *s* and year *t* is

$W_{CD}\frac{C_{t}^{s}}{D_{t}^{s}}$ .

This stratification is conducted for the analysis of the entire population and for sub-analyses related to education, median age, partner status, and nursing home eligibility. Age groups for both the entire population and sub-analyses are determined by six percentile groups. Regarding earnings, we employ four groups for the entire population: one group with no earnings and three groups representing 33.3% percentiles, conditioned on earnings being greater than zero. For our sub-analyses, we utilize six groups for earnings: one group with no earnings and five groups representing 20% percentiles, conditioned on earnings being greater than zero. Earnings plus benefits is categorized into six percentile groups for all analyses. In terms of the source of income, we consider employment status. The corresponding values for all groups are detailed in Table 5.

**Supplemental Table 4. Values groups for sampling weight types**

| **Outcome** | **Base year** | **Age groups** | **Outcome groups** |
| --- | --- | --- | --- |
| **Earnings** |  |  |  |
| Earnings (whole population) | 2003 | 1: [23, 37)  2: [37, 41)  3: [41, 44)  4: [44, 46)  5: [46, 48)  6: [48, 51) | 1: 0  2: [0, 27.527)  3: [27.527, 50.839)  4: [50.839, ∞) |
| Earnings (sub analysis primary education) | 2003 | 1: [23, 35)  2: [35, 40)  3: [40, 43)  4: [43, 46)  5: [46, 48)  6: [48, 51) | 1: 0  2: [0, 19.579)  3: [19.579, 40.062)  4: [40.062, ∞) |
| Earnings (sub analysis secondary education) | 2003 | 1: [23, 34)  2: [34, 39)  3: [39, 42)  4: [42, 45)  5: [45, 47)  6: [47, 51) | 1: 0  2: [0, 24.894)  3: [24.894, 47.582)  4: [47.582, ∞) |
| Earnings (sub analysis higher education) | 2003 | 1: [23, 34)  2: [34, 39)  3: [39, 43)  4: [43, 45)  5: [45, 47)  6: [47, 51) | 1: 0  2: [0, 42.528)  3: [42.528, 71.550)  4: [71.550, ∞) |
| Earnings (sub analysis below median age) | 2003 | 1: [23, 33)  2: [33, 36)  3: [36, 39)  4: [39, 41)  5: [41, 42)  6: [42, 50) | 1: 0  2: [0, 25.191)  3: [25.191, 47.245)  4: [47.245, ∞) |
| Earnings (sub analysis above median age) | 2003 | 1: [42, 45)  2: [45, 46)  3: [46, 47)  4: [47, 48)  5: [48, 49)  6: [49, 51) | 1: 0  2: [0, 30.213)  3: [30.213, 54.351)  4: [54.351, ∞) |
| Earnings (sub analysis with partner) | 2003 | 1: [23, 38)  2: [38, 42)  3: [42, 45)  4: [45, 47)  5: [47, 48)  6: [48, 51) | 1: 0  2: [0, 28.223)  3: [28.883, 54.753)  4: [54.753, ∞) |
| Earnings (sub analysis without partner) | 2003 | 1: [23, 35)  2: [35, 40)  3: [40, 43)  4: [43, 46)  5: [46, 48)  6: [48, 51) | 1: 0  2: [0, 26.329)  3: [26.329, 46.671)  4: [46.671, ∞) |
| Earnings (sub analysis with nursing home eligibility) | 2003 | 1: [23, 39)  2: [39, 43)  3: [43, 45)  4: [45, 47)  5: [47, 49)  6: [49, 51) | 1: 0  2: [0, 26.770)  3: [26.770, 48.979)  4: [48.979, ∞) |
| Earnings (sub analysis without nursing home eligibility) | 2003 | 1: [23, 36)  2: [36, 41)  3: [41, 44)  4: [44, 46)  5: [46, 48)  6: [48, 51) | 1: 0  2: [0, 27.570)  3: [27.570, 51.047)  4: [51.047, ∞) |
| Earnings (sub analysis employed individuals) | 2003 | 1: [23, 35)  2: [35, 39)  3: [39, 43)  4: [43, 44)  5: [44, 46)  6: [46, 51) | 1: 0  2: [0, 32.234)  3: [32.234, 51.958)  4: [51.958, ∞) |
|  |  |  |  |
| **Earnings plus benefits** |  |  |  |
| Earnings plus benefits (whole population) | 2003 | 1: [23, 37)  2: [37, 41)  3: [41, 44)  4: [44, 46)  5: [46, 48)  6: [48, 51) | 1: [0, 12.019)  2: [12.019, 18.567)  3: [18.567, 26.078)  4: [26.078, 35.591)  5: [35.591, 46.549)  6: [46.549, ∞) |
| Earnings plus benefits (sub analysis primary education) | 2003 | 1: [23, 35)  2: [35, 40)  3: [40, 43)  4: [43, 46)  5: [46, 48)  6: [48, 51) | 1: [0, 9.955)  2: [9.955, 16.758)  3: [16.758, 20.261)  4: [20.261, 26.430)  5: [26.430, 36.975)  6: [36.975, ∞) |
| Earnings plus benefits (sub analysis secondary education) | 2003 | 1: [23, 34)  2: [34, 39)  3: [39, 42)  4: [42, 45)  5: [45, 47)  6: [47, 51) | 1: [0, 14.611)  2: [14.611, 20.934)  3: [20.934, 28.261)  4: [28.261, 36.276)  5: [36.276, 44.697)  6: [44.697, ∞) |
| Earnings plus benefits (sub analysis higher education) | 2003 | 1: [23, 34)  2: [34, 39)  3: [39, 43)  4: [43, 45)  5: [45, 47)  6: [47, 51) | 1: [0, 20.537)  2: [20.537, 31.722)  3: [31.722, 42.353)  4: [42.353, 52.341)  5: [52.341, 67.901)  6: [67.901, ∞) |
| Earnings plus benefits (sub analysis below median age) | 2003 | 1: [23, 33)  2: [33, 36)  3: [36, 39)  4: [39, 41)  5: [41, 42)  6: [42, 50) | 1: [0, 12.449)  2: [12.449, 17.815)  3: [17.815, 24.144)  4: [24.144, 32.889)  5: [32.889, 43.383)  6: [43.383, ∞) |
| Earnings plus benefits (sub analysis above median age) | 2003 | 1: [42, 45)  2: [45, 46)  3: [46, 47)  4: [47, 48)  5: [48, 49)  6: [59, 51) | 1: [0, 11.507)  2: [11.507, 19.372)  3: [19.372, 28.162)  4: [28.162, 38.022)  5: [38.022, 49.986)  6: [49.986, ∞) |
| Earnings plus benefits (sub analysis with partner) | 2003 | 1: [23, 38)  2: [38, 42)  3: [42, 45)  4: [45, 47)  5: [47, 48)  6: [48, 51) | 1: [0, 6.764)  2: [6.764, 18.634)  3: [18.634, 29.258)  4: [29.258, 39.673)  5: [39.673, 51.461)  6: [51.461, ∞) |
| Earnings plus benefits (sub analysis without partner) | 2003 | 1: [23, 35)  2: [35, 40)  3: [40, 43)  4: [43, 46)  5: [46, 48)  6: [48, 51) | 1: [0, 16.277)  2: [16.277, 18.562)  3: [18.562, 24.056)  4: [24.056, 31.763)  5: [31.763, 42.133)  6: [42.133, ∞) |
| Earnings plus benefits (sub analysis with nursing home eligibility) | 2003 | 1: [23, 39)  2: [39, 43)  3: [43, 45)  4: [45, 47)  5: [47, 49)  6: [49, 51) | 1: [0, 11.479)  2: [11.479, 17.716)  3: [17.716, 25.056)  4: [25.056, 33.402)  5: [33.402, 44.947)  6: [44.947, ∞) |
| Earnings plus benefits (sub analysis without nursing home eligibility) | 2003 | 1: [23, 36)  2: [36, 41)  3: [41, 44)  4: [44, 46)  5: [46, 48)  6: [48, 51) | 1: [0, 12.182)  2: [12.182, 18.685)  3: [18.685, 26.163)  4: [26.163, 35.807)  5: [35.807, 46.708)  6: [46.708, ∞) |
| Earnings plus benefits (sub analysis employed individuals) | 2003 | 1: [23, 35)  2: [35, 39)  3: [39, 43)  4: [43, 44)  5: [44, 46)  6: [46, 51) | 1: [0, 20.583)  2: [20.583, 28.518)  3: [28.518, 35.939)  4: [35.939, 41.985)  5: [41.985, 52.693)  6: [52.693, ∞) |
|  |  |  |  |
| **Income Source** |  |  |  |
| Income Source (whole population) | 1999 | 1: [19, 33)  2: [33, 37)  3: [37, 40)  4: [40, 42)  5: [42, 44)  6: [44, 47) | 1: No employment  2: Employed |
| Income Source (sub analysis primary education) | 1999 | 1: [19, 32)  2: [32, 36)  3: [36, 39)  4: [39, 41)  5: [41, 44)  6: [44, 47) | 1: No employment  2: Employed |
| Income Source (sub analysis secondary education) | 1999 | 1: [19, 30)  2: [30, 35)  3: [35, 39)  4: [39, 41)  5: [41, 43)  6: [43, 47) | 1: No employment  2: Employed |
| Income Source (sub analysis higher education) | 1999 | 1: [19, 30)  2: [30, 35)  3: [35, 39)  4: [39, 41)  5: [41, 43)  6: [43, 47) | 1: No employment  2: Employed |
| Income Source (sub analysis below median age) | 1999 | 1: [19, 29)  2: [29, 32)  3: [32, 35)  4: [35, 37)  5: [37, 39)  6: [39, 46) | 1: No employment  2: Employed |
| Income Source (sub analysis above median age) | 1999 | 1: [38, 41)  2: [41, 42)  3: [42, 43)  4: [43, 44)  5: [44, 45)  6: [45, 47) | 1: No employment  2: Employed |
| Income Source (sub analysis with partner) | 1999 | 1: [19, 34)  2: [34, 38)  3: [38, 41)  4: [41, 43)  5: [43, 44)  6: [44, 47) | 1: No employment  2: Employed |
| Income Source (sub analysis without partner) | 1999 | 1: [19, 31)  2: [31, 36)  3: [36, 39)  4: [39, 42)  5: [42, 44)  6: [44, 47) | 1: No employment  2: Employed |
| Income Source (sub analysis nursing home eligibility) | 1999 | 1: [19, 35)  2: [35, 39)  3: [39, 41)  4: [41, 43)  5: [43, 45)  6: [45, 47) | 1: No employment  2: Employed |
| Income Source (sub analysis without nursing home eligibility) | 1999 | 1: [19, 32)  2: [32, 37)  3: [37, 40)  4: [40, 42)  5: [42, 44)  6: [44, 47) | 1: No employment  2: Employed |
| Income Source (sub analysis employed individuals) | 1999 | 1: [19, 31)  2: [31, 35)  3: [35, 39)  4: [39, 40)  5: [40, 42)  6: [42, 47) | 1: No employment  2: Employed |

**Appendix C – Outcome measures**

**Supplemental Table 5. Definition outcome measures – two income types**

| **Income type** | **Definition** |
| --- | --- |
| Earnings | Earnings include an individual's gross income from employment and from their own business.   1. Income from employment consists of the gross salary (including both employee and employer contributions to social insurance premiums), bonuses, and compensation for labor performed outside of an employment relationship. It also includes fringe benefits such as the value of the private use of an employer's car. 2. Income from own business represents the compensation for self-employed individuals for their labor and business assets. |
| Earnings plus benefits | Earnings plus benefits include the following components of an individual's gross income:   1. Income from employment 2. Income from own business 3. Income insurance benefits 4. Social assistance (excluding child benefit and child tax credit). |

**Supplemental Table 6. Definition outcome measures – income sources**

| **Income sources** | **Description** |
| --- | --- |
| Employment | Income from employment concerns:   1. Employees: Individuals who, in the reporting year, are either employed in the Netherlands or earn income from work abroad and are not actively engaged in a main position as a director-major shareholder. Data on income from work abroad is not available for the reporting years 1999 and 2000 and is therefore only included from 2001 onwards. 2. Director-major shareholders: Individuals who, in the reporting year, hold a position as a director-major shareholder. 3. Self-employed entrepreneurs: Individuals who, in the reporting year, have profit from a business. 4. Other self-employed persons: Individuals who, in the reporting year, have income from other employment. 5. Contributing family members: Individuals who, in the reporting year, are active as contributing partners of a self-employed entrepreneur. |
| Disability Insurance Benefit | Individuals who received sickness or disability benefits in the reporting year. Sickness and disability benefits include:   1. Sickness Benefits Act (ZW) 2. Disablement Insurance Act (WAO) benefits 3. Self-Employed Persons Disablement Benefits Act (Waz) benefits 4. Supplementary Benefits Act (TW in combination with disability benefits). For individuals already receiving disability insurance benefits whose circumstances change, leading to income falling below the social minimum. 5. Work and Income (Capacity for Work) Act (WIA) benefits, including the Fully Disabled Persons Income Support Scheme (IVA) and the Partially Disabled Persons Return to Work Scheme (WGA). |
| Welfare Benefit | Individuals who received welfare benefits in the reporting year. Welfare benefits include:   1. Benefits under the General Assistance Act (ABW) 2. Benefits under the Work and Social Assistance Act (WWB). |
| Unemployment Benefit | Individuals who received unemployment benefits in a reporting year. Unemployment benefits include:   1. Benefits under the Unemployment Insurance Act (WW) 2. Government severance payments 3. Benefits under the Supplementary Benefits Act (TW in combination with WW benefits). For individuals already receiving unemployment benefits whose circumstances change, leading to income falling below the social minimum. |
| Retirement Benefit | Individuals who received a pension in the reporting year. Pension payments include:   1. General Old Age Pension Act (AOW) benefits 2. General Surviving Relatives Act (Anw) benefits 3. Other pensions and annuities 4. Income from foreign pensions (from the reporting year 2001 onwards) |
| Social Security Other | Individuals who received benefits from other social assistance in a reporting year. Other social assistance include:   1. Benefits under the Older and Partially Disabled Unemployed Workers Income Support Act (IOAW) 2. Benefits under the Older and Partially Disabled Former Self-Employed Persons Income Support Act (IOAZ) 3. Benefits under the Decree on Assistance to Self-Employed Persons (Bbz) 4. Benefits under the Work and Income for Artists Act (WWIK) 5. Benefits under the Disability Provisions for Young Disabled Persons Act (Wajong) 6. War and resistance pensions 7. Other unspecified benefits |

**Appendix D – Demographic and socioeconomic characteristics**

**Supplemental Table 7. Classification of education levels**

| **Education level** | **Definition** |
| --- | --- |
| Primary education | - Primary education group 1-2 - Primary education group 3-8 - Practical training - Vmbo-b/k - Mbo1 - Vmbo-g/t - Havo-, vwo year 1-3 |
| Secondary education | - Mbo2 - Mbo3 - Mbo4 - Havo year 4-5 - Vwo year 4-6 |
| Higher education | - Hbo-associate degree - Hbo-bachelor - Wo-bachelor - Hbo-master - Wo-master - Doctorate |

**Supplemental Table 8. Average sample characteristics by Ever-Dementia status (not weighted)**

|  | **Dementia cases (n=16,010)** | | **Controls (n=129,616)** | |
| --- | --- | --- | --- | --- |
|  | **2003** | **2016** | **2003** | **2016** |
| **Characteristics** |  |  |  |  |
| Age, mean [SD] | **42.5**  **[5.9]** | **55.4**  **[6.0]** | **36.7**  **[8.3]** | **49.6**  **[8.3]** |
| Female, % | **45.5** | **45.3** | **50.2** | **50.5** |
| Western, % | **87.7** | **86.7** | **90.5** | **89.1** |
| w/ Partner, % | **54.9** | **46.7** | **58.3** | **63.2** |
| Education level, % |  |  |  |  |
| *Primary education* | **24.1** | **25.0** | **12.3** | **12.9** |
| *Secondary education* | **19.1** | **18.9** | **20.4** | **20.3** |
| *Higher education* | **10.2** | **10.0** | **21.4** | **20.8** |
| *Missing value* | **46.5** | **46.1** | **45.9** | **46.0** |
|  |  |  |  |  |
| Annual earnings in EUR, mean [Standard Deviation] | **28,906**  **[32,402]** | **15,717**  **[28,121]** | **39,625**  **[33,589]** | **42,029**  **[38,714]** |
| Annual earnings plus benefits in EUR, mean [Standard Deviation] | **30,190**  **[22.674]** | **28.009**  **[22.383]** | **34,672**  **[25,715]** | **40,315**  **[30,940]** |
|  |  |  |  |  |
|  |  |  |  |  |
|  | **1999** | **2016** | **1999** | **2016** |
| Employment, % | **67.0** | **37.5** | **80.1** | **79.3** |
| Disability Insurance Benefit, % | **17.3** | **37.4** | **6.8** | **9.0** |
| Welfare Benefit, % | **13.8** | **17.0** | **4.8** | **5.3** |
| Unemployment Benefit, % | **4.9** | **6.7** | **3.5** | **7.4** |
| Retirement Benefit, % | **0.7** | **21.4** | **0.3** | **12.1** |
| Social Security Other, % | **5.1** | **10.9** | **1.8** | **3.1** |

**Appendix E - Explanation statistical analysis non-parametric event study**

For earnings and earnings plus benefits, we included yearly indicator variables ranging from 17 years prior to and 1 year after the year dementia was first identified, comparing them with a control group of individuals below the age of 65 without dementia in the observed period. The same approach was applied to income sources, with the yearly indicator variables spanning from 21 years before identification to 1 year after identification. The basic non-parametric event study specification takes the following form:

$Y_{it}=\alpha+\sum_{k=-K}^{1} \begin{aligned} 1\left[ K_{it}=k \right]\beta_{k} \end{aligned}+X_{it}\gamma+\delta_{t}+\varepsilon_{it}$,

where $Y_{it}$ is the outcome – earnings, earnings plus benefits, and income sources – for individual *i* in calendar year *t*. $1[K_{it}=k]$ is an indicator function equal to 1 if the observation is *k* years from the identification of dementia. *k* spans from *K* years prior to the identification of dementia up to 1 year after (*k* = 1). Specifically, K is set at 17 for earnings and earnings plus benefits, and K is set at 21 for income sources. For the control group, all time-to-event indicator dummies are zero. The coefficients $\beta_{k}$are the main coefficients of interest as they measure the difference in outcomes compared to the dementia identification year, relative to the control group. $X_{it}\gamma$represents a vector for with control variables: age, gender, migrant status, partner status, education categories, ever dementia status, and $\delta_{t}$ calendar year indicators. Finally, $\varepsilon_{it}$ is the individual- and time-specific error term, which is clustered at the individual level. We use Wald tests to compare coefficients to the coefficient for the earliest period to reveal when the change starts.

**Appendix F – Outcome measures over time (calendar years and event years)**

**Supplemental Table 9. Earnings and Earnings plus benefits in euros over calendar years (individuals in dementia identification year 2020 vs control group)**

| **Year** | **Earnings (dementia 2020 group)** | **Earnings (control group)** | **Earnings plus benefits (dementia 2020 group)** | **Earnings plus benefits (control group)** |
| --- | --- | --- | --- | --- |
| 2003 | 30,502 | 30,463 | 30,832 | 30,803 |
| 2004 | 30,155 | 31,714 | 30,548 | 31,593 |
| 2005 | 29,995 | 32,532 | 30,316 | 31,601 |
| 2006 | 30,978 | 34,159 | 31,238 | 32,991 |
| 2007 | 31,549 | 35,376 | 32,091 | 34,248 |
| 2008 | 31,454 | 35,975 | 32,458 | 35,110 |
| 2009 | 30,511 | 35,905 | 32,619 | 35,625 |
| 2010 | 29,693 | 35,553 | 32,165 | 35,554 |
| 2011 | 28,710 | 35,185 | 31,861 | 35,391 |
| 2012 | 27,113 | 34,347 | 30,934 | 34,813 |
| 2013 | 25,592 | 33,043 | 30,346 | 34,341 |
| 2014 | 24,191 | 32,406 | 29,885 | 34,251 |
| 2015 | 22,656 | 31,532 | 29,392 | 34,273 |
| 2016 | 21,499 | 31,314 | 29,052 | 34,576 |
| 2017 | 20,458 | 30,957 | 28,781 | 34,736 |
| 2018 | 19,259 | 31,263 | 28,542 | 34,635 |
| 2019 | 16,993 | 31,758 | 28,062 | 34,873 |
| 2020 | 14,023 | 31,999 | 27,771 | 35,108 |
| 2021 | 10,673 | 32,746 | 27,193 | 35,786 |

**Supplemental Table 10. Regression results earnings and earnings plus benefits (coefficient plot earnings and earnings plus benefits)**

|  | (1) | (2) |
| --- | --- | --- |
| VARIABLES | Earnings | Earnings plus benefits |
|  |  |  |
| K = -17 | 17,521.93*** | 7,051.61*** |
|  | (16,161.44 - 18,882.42) | (6,093.20 - 8,010.02) |
| K = -16 | 16,642.59*** | 6,302.20*** |
|  | (15,639.98 - 17,645.20) | (5,601.77 - 7,002.63) |
| K = -15 | 15,313.71*** | 5,730.79*** |
|  | (14,491.06 - 16,136.35) | (5,161.64 - 6,299.93) |
| K = -14 | 14,245.91*** | 5,237.67*** |
|  | (13,619.59 - 14,872.22) | (4,814.33 - 5,661.01) |
| K = -13 | 13,564.16*** | 4,868.89*** |
|  | (13,005.23 - 14,123.10) | (4,498.46 - 5,239.32) |
| K = -12 | 12,561.25*** | 4,277.46*** |
|  | (12,023.14 - 13,099.37) | (3,924.25 - 4,630.67) |
| K = -11 | 11,728.08*** | 3,875.47*** |
|  | (11,200.95 - 12,255.20) | (3,529.62 - 4,221.32) |
| K = -10 | 10,911.57*** | 3,463.15*** |
|  | (10,395.87 - 11,427.27) | (3,131.92 - 3,794.38) |
| K = -9 | 10,047.94*** | 3,066.06*** |
|  | (9,544.09 - 10,551.80) | (2,740.21 - 3,391.92) |
| K = -8 | 9,361.99*** | 2,891.05*** |
|  | (8,871.08 - 9,852.91) | (2,577.01 - 3,205.09) |
| K = -7 | 8,696.40*** | 2,688.59*** |
|  | (8,226.24 - 9,166.55) | (2,386.47 - 2,990.71) |
| K = -6 | 7,780.74*** | 2,425.92*** |
|  | (7,333.36 - 8,228.12) | (2,135.14 - 2,716.70) |
| K = -5 | 6,855.24*** | 2,115.07*** |
|  | (6,434.30 - 7,276.18) | (1,834.50 - 2,395.65) |
| K = -4 | 5,924.53*** | 1,679.39*** |
|  | (5,541.20 - 6,307.85) | (1,411.74 - 1,947.05) |
| K = -3 | 4,866.59*** | 1,370.52*** |
|  | (4,520.15 - 5,213.03) | (1,111.05 - 1,629.99) |
| K = -2 | 3,482.36*** | 930.17*** |
|  | (3,197.98 - 3,766.74) | (685.61 - 1,174.73) |
| K = -1 | 1,876.74*** | 421.87*** |
|  | (1,667.75 - 2,085.73) | (199.86 - 643.88) |
| K = 1 | -2,495.36*** | -546.30*** |
|  | (-2,746.15 - -2,244.57) | (-816.85 - -275.74) |
| Constant | 11,327.24*** | 17,963.98*** |
|  | (10,161.80 - 12,492.68) | (17,215.51 - 18,712.46) |
|  |  |  |
| Observations | 2,643,302 | 2,643,302 |
| R-squared | 0.24 | 0.22 |
| Robust ci in parentheses | |  |
| *** p<0.01, ** p<0.05, * p<0.1 | | |

**Supplemental Table 11. Regression results income sources (coefficient plot income sources)**

|  | (1) | (2) | (3) | (4) | (5) | (6) |
| --- | --- | --- | --- | --- | --- | --- |
| VARIABLES | Employment | Disability Insurance Benefit | Welfare Benefit | Unemployment Benefit | Retirement Benefit | Social Security Other |
|  |  |  |  |  |  |  |
| K = -21 | 0.355*** | -0.243*** | -0.038*** | 0.009** | -0.026*** | -0.036*** |
|  | (0.335 - 0.375) | (-0.260 - -0.226) | (-0.053 - -0.024) | (0.000 - 0.019) | (-0.033 - -0.019) | (-0.047 - -0.026) |
| K = -20 | 0.339*** | -0.239*** | -0.030*** | 0.011*** | -0.027*** | -0.034*** |
|  | (0.324 - 0.353) | (-0.252 - -0.226) | (-0.041 - -0.020) | (0.004 - 0.018) | (-0.034 - -0.020) | (-0.041 - -0.026) |
| K = -19 | 0.321*** | -0.227*** | -0.031*** | 0.013*** | -0.027*** | -0.029*** |
|  | (0.309 - 0.334) | (-0.238 - -0.216) | (-0.040 - -0.022) | (0.007 - 0.020) | (-0.033 - -0.021) | (-0.036 - -0.022) |
| K = -18 | 0.317*** | -0.214*** | -0.030*** | 0.015*** | -0.027*** | -0.029*** |
|  | (0.307 - 0.327) | (-0.223 - -0.205) | (-0.037 - -0.023) | (0.009 - 0.020) | (-0.033 - -0.020) | (-0.035 - -0.024) |
| K = -17 | 0.304*** | -0.215*** | -0.033*** | 0.012*** | -0.027*** | -0.030*** |
|  | (0.294 - 0.313) | (-0.223 - -0.206) | (-0.039 - -0.027) | (0.007 - 0.018) | (-0.033 - -0.020) | (-0.035 - -0.025) |
| K = -16 | 0.287*** | -0.206*** | -0.034*** | 0.014*** | -0.026*** | -0.029*** |
|  | (0.277 - 0.296) | (-0.214 - -0.198) | (-0.040 - -0.028) | (0.008 - 0.019) | (-0.032 - -0.020) | (-0.035 - -0.024) |
| K = -15 | 0.271*** | -0.200*** | -0.034*** | 0.017*** | -0.026*** | -0.031*** |
|  | (0.262 - 0.281) | (-0.208 - -0.192) | (-0.040 - -0.028) | (0.011 - 0.022) | (-0.032 - -0.020) | (-0.036 - -0.025) |
| K = -14 | 0.248*** | -0.194*** | -0.033*** | 0.019*** | -0.026*** | -0.031*** |
|  | (0.239 - 0.257) | (-0.202 - -0.186) | (-0.039 - -0.027) | (0.014 - 0.025) | (-0.032 - -0.020) | (-0.036 - -0.025) |
| K = -13 | 0.235*** | -0.192*** | -0.033*** | 0.021*** | -0.026*** | -0.030*** |
|  | (0.226 - 0.244) | (-0.200 - -0.184) | (-0.038 - -0.027) | (0.016 - 0.027) | (-0.033 - -0.020) | (-0.035 - -0.024) |
| K = -12 | 0.223*** | -0.187*** | -0.033*** | 0.020*** | -0.026*** | -0.026*** |
|  | (0.214 - 0.232) | (-0.195 - -0.179) | (-0.038 - -0.027) | (0.014 - 0.025) | (-0.033 - -0.020) | (-0.031 - -0.021) |
| K = -11 | 0.212*** | -0.183*** | -0.031*** | 0.021*** | -0.025*** | -0.025*** |
|  | (0.203 - 0.221) | (-0.191 - -0.175) | (-0.036 - -0.026) | (0.015 - 0.026) | (-0.032 - -0.019) | (-0.030 - -0.019) |
| K = -10 | 0.204*** | -0.176*** | -0.031*** | 0.017*** | -0.017*** | -0.018*** |
|  | (0.195 - 0.213) | (-0.184 - -0.169) | (-0.036 - -0.025) | (0.011 - 0.022) | (-0.024 - -0.010) | (-0.024 - -0.013) |
| K = -9 | 0.187*** | -0.167*** | -0.027*** | 0.015*** | -0.004 | -0.017*** |
|  | (0.179 - 0.196) | (-0.175 - -0.160) | (-0.031 - -0.022) | (0.009 - 0.021) | (-0.011 - 0.004) | (-0.022 - -0.011) |
| K = -8 | 0.174*** | -0.161*** | -0.024*** | 0.011*** | 0.010*** | -0.012*** |
|  | (0.165 - 0.182) | (-0.169 - -0.154) | (-0.028 - -0.019) | (0.006 - 0.017) | (0.003 - 0.018) | (-0.017 - -0.007) |
| K = -7 | 0.158*** | -0.154*** | -0.023*** | 0.010*** | 0.013*** | -0.009*** |
|  | (0.150 - 0.166) | (-0.161 - -0.147) | (-0.027 - -0.018) | (0.004 - 0.015) | (0.006 - 0.021) | (-0.014 - -0.004) |
| K = -6 | 0.142*** | -0.138*** | -0.022*** | 0.009*** | 0.015*** | -0.007*** |
|  | (0.134 - 0.150) | (-0.145 - -0.132) | (-0.026 - -0.018) | (0.003 - 0.015) | (0.008 - 0.022) | (-0.012 - -0.003) |
| K = -5 | 0.125*** | -0.127*** | -0.018*** | 0.007** | 0.017*** | -0.005** |
|  | (0.118 - 0.133) | (-0.133 - -0.120) | (-0.022 - -0.014) | (0.001 - 0.013) | (0.010 - 0.024) | (-0.009 - -0.001) |
| K = -4 | 0.110*** | -0.112*** | -0.017*** | 0.004 | 0.017*** | -0.003 |
|  | (0.103 - 0.117) | (-0.119 - -0.106) | (-0.020 - -0.013) | (-0.001 - 0.010) | (0.010 - 0.023) | (-0.007 - 0.001) |
| K = -3 | 0.089*** | -0.094*** | -0.014*** | 0.004 | 0.016*** | -0.001 |
|  | (0.082 - 0.095) | (-0.100 - -0.088) | (-0.017 - -0.010) | (-0.001 - 0.009) | (0.010 - 0.022) | (-0.005 - 0.002) |
| K = -2 | 0.063*** | -0.073*** | -0.010*** | 0.004* | 0.016*** | 0.001 |
|  | (0.058 - 0.068) | (-0.078 - -0.068) | (-0.013 - -0.007) | (-0.001 - 0.009) | (0.011 - 0.022) | (-0.003 - 0.004) |
| K = -1 | 0.034*** | -0.043*** | -0.007*** | 0.003 | 0.010*** | 0.001 |
|  | (0.030 - 0.038) | (-0.047 - -0.039) | (-0.009 - -0.005) | (-0.001 - 0.007) | (0.005 - 0.015) | (-0.002 - 0.003) |
| K = 1 | -0.051*** | 0.065*** | 0.004*** | -0.004* | -0.017*** | 0.001 |
|  | (-0.056 - -0.046) | (0.059 - 0.070) | (0.001 - 0.007) | (-0.009 - 0.000) | (-0.022 - -0.012) | (-0.002 - 0.004) |
| Constant | 0.605*** | 0.104*** | 0.102*** | -0.044*** | -0.020*** | 0.034** |
|  | (0.558 - 0.652) | (0.069 - 0.140) | (0.073 - 0.132) | (-0.050 - -0.037) | (-0.024 - -0.016) | (0.006 - 0.063) |
|  |  |  |  |  |  |  |
| Observations | 3,244,081 | 3,244,081 | 3,244,081 | 3,244,081 | 3,244,081 | 3,244,081 |
| R-squared | 0.182 | 0.084 | 0.194 | 0.045 | 0.125 | 0.038 |
| Robust ci in parentheses | |  |  |  |  |  |
| *** p<0.01, ** p<0.05, * p<0.1 | | |  |  |  |  |
